# First-principles study of novel Cs_2_LuCoH_6_ and Cs_2_LuZnH_6_ double hydride perovskites for hydrogen storage applications

**DOI:** 10.1039/d6ra02417c

**Published:** 2026-05-28

**Authors:** Rasmiah S. Almufarij, Malik Shafqat Hayat, R. M. Arif Khalil, Shoug M. Alghamdi, Elsammani Ali Shokralla, Jack Arayro, Mohamed Abdelsabour Fahmy, Mohamed A. Siddig, Ahmed Samir, Arslan Ashfaq

**Affiliations:** a Department of Chemistry, College of Science, Princess Nourah Bint Abdulrahman University P. O. Box 84428 Riyadh 11671 Saudi Arabia; b Department of Physics, Emerson University Multan 60000 Pakistan arslan.ashfaq201@gmail.com; c Institute of Physics, Bahauddin Zakariya University Multan 60800 Pakistan; d Department of Physics, College of Science in Yanbu, Taibah University Yanbu Governorate Saudi Arabia; e Department of Physics, Faculty of Science, Al-Baha University Alaqiq 65779-7738 Saudi Arabia; f College of Engineering and Technology, American University of the Middle East Egaila 54200 Kuwait; g Department of Mathematics, Adham University College, Umm Al-Qura University Adham Makkah 28653 Saudi Arabia; h Department of Basic Sciences, Faculty of Computers and Informatics, Suez Canal University, New Campus Ismailia 41522 Egypt; i Physics Department, Faculty of Science and Arts, King Khalid University Muhayil Asir Saudi Arabia; j Department of Physics, College of Science, University of Bahri Sudan

## Abstract

This work presents a comprehensive evaluation of the structural, electronic, vibrational, magnetic, and mechanical characteristics of the double hydride perovskites Cs_2_LuCoH_6_ and Cs_2_LuZnH_6_, with the aim of determining their potential for hydrogen storage and related energy applications. The fully relaxed and optimized structures exhibit negative cohesive energies of −3.57 eV per atom for Cs_2_LuCoH_6_ and −2.65 eV per atom for Cs_2_LuZnH_6_, confirming their thermodynamic stability. Electronic band structure analysis reveals semiconductor behavior with an energy band gap of 0.917 eV for Cs_2_LuCoH_6_, while Cs_2_LuZnH_6_ displays metallic behavior with a zero band gap. Phonon dispersion calculations confirm the dynamic stability of Cs_2_LuCoH_6_, showing no imaginary modes, whereas Cs_2_LuZnH_6_ exhibits a few negative phonon frequencies, indicating partial instability. Magnetic analysis demonstrates a ferromagnetic phase with an overall magnetic moment of 1.25 µB for Cs_2_LuCoH_6_ and nonmagnetic behavior for Cs_2_LuZnH_6_. The computed hydrogen storage capacities (by weight) are 4.87 wt% for Cs_2_LuCoH_6_ and 4.46 wt% for Cs_2_LuZnH_6_. The tolerance factors (0.92 for Cs_2_LuCoH_6_ and 0.84 for Cs_2_LuZnH_6_) further confirm the structural symmetry and mechanical robustness of these compounds. These DFT-based results suggest that Cs_2_LuCoH_6_ and Cs_2_LuZnH_6_ are promising and novel candidates for use in next-generation hydrogen storage devices and energy-related applications.

## Abstract 2

DFT study of Cs_2_LuCoH_6_ and Cs_2_LuZnH_6_ hydride perovskites shows Co-based compound is stable ferromagnetic semiconductor with good hydrogen storage potential, while Zn-based is metallic and partially unstable, highlighting tunable energy applications.

## Graphical abstract

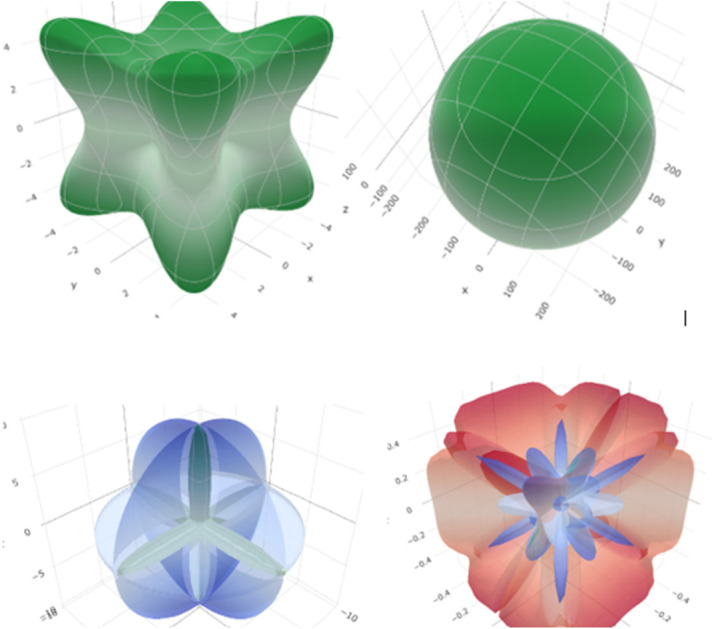

## Introduction

In the current time, the ultimate challenge is to achieve progress in the field of the development of energy systems that are environmentally friendly, using renewable, sustainable energy sources to overcome our dependency on fossil fuels. Due to the burning of fossil fuels, nitrogen oxides and carbon dioxide, along with other harmful particles, are released. Due to these factors, climate change and local extreme air pollution are observed globally.^1,2^

Attaining the Sustainable Development Goals (SDGs) affects food conservation, the enhancement of human well-being and the management of waste in several forms. According to the seventh global goal (SDG7), energy is essential for widespread access to reliable, sustainable, contemporary and economical energy sources.^3,4^ Three key benchmarks for achieving SDG 7 (Affordable and Clean Energy) at the international level are: (i) increasing the share of renewable and green energy in the global energy mix; (ii) improving energy efficiency by maximizing the effective use of all generated energy; and (iii) ensuring universal access to reliable, affordable, and modern energy services for all. Nowadays, research is focused both theoretically and experimentally on producing clean energy and creating efficient carriers to accomplish sustainable civilization. Regarding the energy transition, technologies for energy generation (acquisition), storage, and transportation have attracted significant global attention.^5,6^

Hydrogen, in fact, has been observed as an excellent alternative energy carrier compared to fossil fuels such as natural gas, coal and oil. However, the development of hydrogen as an energy carrier presents significant hurdles, especially concerning its storage. Hydrogen can be effectively and safely stored using hydride perovskites.^7–9^ Materials for hydrogen storage purposes must satisfy some common requirements, such as large volumetric and gravimetric hydrogen storage capacities, ambient conditions for hydrogen release, good kinetics, *etc.* Perovskites are referred to as materials with these unique chemical and physical characteristics. Due to the vast variety of material structures, a wide range of physicochemical properties has been observed for the perovskite family.^10–12^ Perovskite-based structures deliver a large surface area for hydrogen adsorption and can be adapted for maximum hydrogen adsorption. In such materials, a large variety of physical characteristics has been observed. As concerns hydrogen storage, perovskite hydrides are ideal. The simulation of solid-state hydrogen storage compounds, particularly ABH_3_ hydride perovskites, has exposed various compounds for hydrogen storage.^13^ It is crucial to highlight that simulations of various compounds have been carried out, and many of them have gained popularity as hydrogen storage materials, such as (Ca/Sr)CuH_3_, NaMgH_3_, (Rb/Cs)InH_3_, and (Rb/Cs)BH_3_, along with the unique perovskite hydrides (Rb/Cs)_2_CaH_4_ (X = Ba, Sr, Cs) and (Sr/Ba)NiH_3_. Furthermore, first-principles simulations offer an economical and valuable technique to study and locate ideal H_2_ storage compounds.^14^ According to current knowledge, double-hydride perovskites have hardly been investigated experimentally or theoretically. The structure of double-hydride perovskites is a three-dimensional system with positive cations surrounded by octahedral structures. In the A_2_BXH_6_ structure, hydrogen (H) acts as an anion (hydride). The B-site is typically occupied by a transition metal, while the X-site is an alkali metal. The A-site is filled by a positively charged cation, which helps stabilize the overall crystal structure.^15^ Our research group has simulated a large number of hydride perovskite materials for hydrogen storage applications. The XCuH_3_ (X = Ni, Co, Zn) series represents a class of hydride perovskites with promising hydrogen storage capability. The gravimetric hydrogen storage capacities are calculated to be 3.0 wt% for NiCuH_3_, 2.8 wt% for CoCuH_3_, and 2.7 wt% for ZnCuH_3_, indicating slight variations depending on the transition metal substitution at the X-site. Moreover, metallic character is observed for all these materials.^16^ A series of LiXH_3_ (X = Cr, Co, Fe, Zn) materials was simulated by our research group. These are unique materials for hydrogen storage and related applications.^17^ In addition, Cs_2_CaTlH_6_, Cs_2_SrTlH_6_, and Cs_2_BaTlH_6_ are another class of hydride materials with novel and significant physicochemical properties that make these materials favorable for hydrogen (H) energy storage systems.^18^ Importantly, recent advances have demonstrated a growing interest in hydride-based and double perovskite systems;^19,20^ however, most reported studies focus on conventional transition-metal or alkaline-earth systems, while rare-earth (lanthanide)-based double hydride perovskites remain largely unexplored. In particular, the role of Lu-based double hydride perovskites combined with transition metals has not been systematically investigated for hydrogen storage applications, despite their potential to exhibit tunable electronic structures, magnetic behavior, and enhanced stability. Motivated by this research gap, we propose two novel double hydride perovskites, Cs_2_LuCoH_6_ and Cs_2_LuZnH_6_, for a comprehensive first-principles investigation.

In this article, we examine the properties of Cs_2_LuCoH_6_ and Cs_2_LuZnH_6_ double hydride compounds for H energy storage and related applications. DFT-based simulations are calculated using the CASTEP simulation code and by utilizing the hybrid complex HSE06 functional. The first section of the present article consists of an introduction to hydride perovskites, the second section illustrates the DFT-based methodology, and finally, the third section provides the results and discussion about the Cs_2_LuCoH_6_ and Cs_2_LuZnH_6_ double hydride perovskites. From these DFT-based calculations, it has been illustrated that these Cs_2_LuCoH_6_ and Cs_2_LuZnH_6_ double hydride compounds have the potential to revolutionize the field and motivate research into synthesizing these compounds for hydrogen energy storage systems. The present study highlights that these novel double hydride perovskites exhibit promising multifunctional properties, demonstrating superior stability and potential for hydrogen storage applications.

### Research methodology

Density functional theory (DFT) is a powerful first-principles approach widely used to investigate the structural, electronic, vibrational, and mechanical properties of materials. In this study, all calculations involving the double hydride perovskites Cs_2_LuCoH_6_ and Cs_2_LuZnH_6_ were performed using the CASTEP code within the framework of plane-wave pseudopotential DFT. The electronic exchange–correlation effects were treated using the generalized gradient approximation (GGA) in the form of Perdew–Burke–Ernzerhof (PBE) for structural optimization, while the HSE06 hybrid functional was employed to obtain more accurate electronic band structures. The Kohn–Sham equations were solved self-consistently using a plane-wave basis set. Structural optimizations were carried out using the Broyden–Fletcher–Goldfarb–Shanno (BFGS) minimization algorithm until convergence was achieved.^21^ The convergence criteria were set to a total energy tolerance of 5 × 10^−6^ eV per atom, maximum force of 0.01 eV Å^−1^, maximum stress of 0.02 GPa, and maximum atomic displacement of 5 × 10^−4^ Å, ensuring highly accurate geometry optimization. A plane-wave cutoff energy of 520 eV was used for all calculations.^22,23^ The Brillouin zone was sampled using a Monkhorst–Pack *k*-point grid of appropriate density (optimized for convergence tests) to ensure numerical accuracy in total energy and electronic structure calculations. The phonon dispersion and vibrational stability were evaluated using the finite displacement (supercell) method, as implemented in CASTEP. A 1 × 2 × 1 supercell was constructed to calculate interatomic force constants and phonon spectra, which were used to assess the dynamical stability of the investigated materials.^24^ The dielectric and optical properties were analyzed using the Kramers–Kronig relations, which relate to the real and imaginary parts of the dielectric function and describe the interaction between electromagnetic radiation and the material.^25–27^ All structural relaxations were performed using the conjugate gradient method, and self-consistency was achieved when energy changes between successive iterations were less than the defined convergence threshold.^28^ These computational parameters ensure reliable and reproducible predictions of the structural, electronic, vibrational, and mechanical properties of Cs_2_LuCoH_6_ and Cs_2_LuZnH_6_ for hydrogen storage applications.

## Results and discussion

**Fig. 1:**
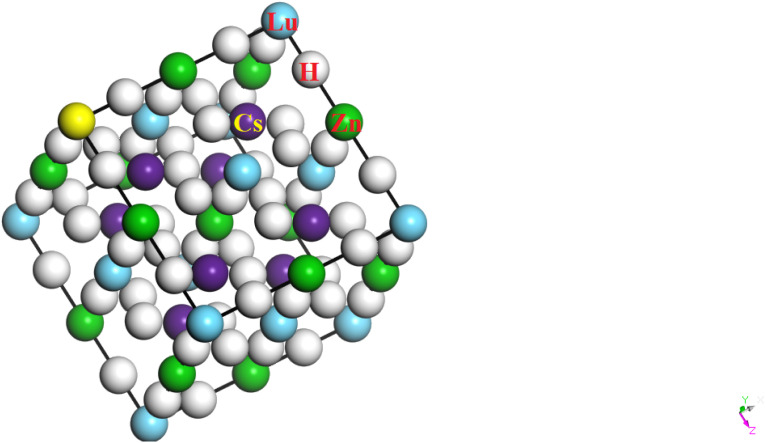
The relaxed and energy-minimized crystal structure of Cs_2_Lu(Co, Zn)H_6_ double hydride perovskite.

Fig. 1 presents the relaxed, stable and energy-minimized crystal structure of Cs_2_LuCoH_6_ and Cs_2_LuZnH_6_ double hydride perovskites with a cubic crystal structure. In the equilibrium structure obtained after relaxation, X = Co, Zn atoms are positioned in face-centered (1/2, ½, ½) positions, Cs atoms are positioned in body-centered positions, Lu atoms are located at corner sites, and H is fixed at face-centered positions with coordinates (1/4, ¼, ¼) in the optimized crystal structure of the materials under consideration. The geometries of these compounds were optimized and structurally relaxed by utilizing the DFT-based hybrid complex HSE06 hybrid functional, as implemented in the CASTEP simulation package. The point group P_1_ (32: oh, *m*3̄*m*, 4/m–32/m) and space group *C*_1_ (225: *Fm*3̄*m*, −*F* 423) are found for the Cs_2_LuCoH_6_ and Cs_2_LuZnH_6_ double hydride perovskites. The supercell structures of Cs_2_LuCoH_6_ and Cs_2_LuZnH_6_ consist of 10 atoms associated with 6 hydride (H^−^) ions per formula unit, forming the extended crystal lattice of these materials. The relaxed structure contains a total of four atomic species, and the optimized supercell uses a maximum of four *k*-points in the Brillouin zone sampling. The geometrical optimization and relaxation of the single-point crystal structure are simulated by the variational principle. The calculated total energies of the perovskites show that Cs_2_LuCoH_6_ has a lower energy value (−7167.43 eV) compared to Cs_2_LuZnH_6_ (−7067.53 eV), indicating that Cs_2_LuCoH_6_ is energetically more stable than Cs_2_LuZnH_6_. The negative values of the total energy predict the chemical, thermodynamic, and structural stability and reliability of the Cs_2_LuCoH_6_ and Cs_2_LuZnH_6_ materials.

Another novel structural parameter is the cohesive energy that illustrates the properties of the Cs_2_LuCoH_6_ and Cs_2_LuZnH_6_ double hydride perovskite materials. The binding energy is actually the energy needed to separate the constituent atoms from the corresponding material and is given by the following expression:^29^1
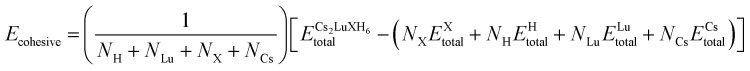
In the above equation, *N*_Cs_, *N*_X_, *N*_Lu_, and *N*_H_ depict the numbers of Cs, X (Co or Zn), Lu and H atoms within the Cs_2_LuXH_6_ (X = Co or Zn) double hydride perovskite material within the unit cell of the structure. The cohesive energies, calculated from the ground-state energies, are found to be negative in all cases, with deviations observed in their magnitudes; for example, they are −3.57 eV per atom for Cs_2_LuCoH_6_ and −2.65 eV per atom for Cs_2_LuZnH_6_. Finally, the structural reliability, stability and compatibility of the double hydrides Cs_2_LuCoH_6_ and Cs_2_LuZnH_6_ are verified owing to the consistently negative values of the cohesive energies. These structural properties confirmed that Cs_2_LuCoH_6_ and Cs_2_LuZnH_6_ are potentially transformative materials for H storage systems and associated applications.

**Fig. 2:**
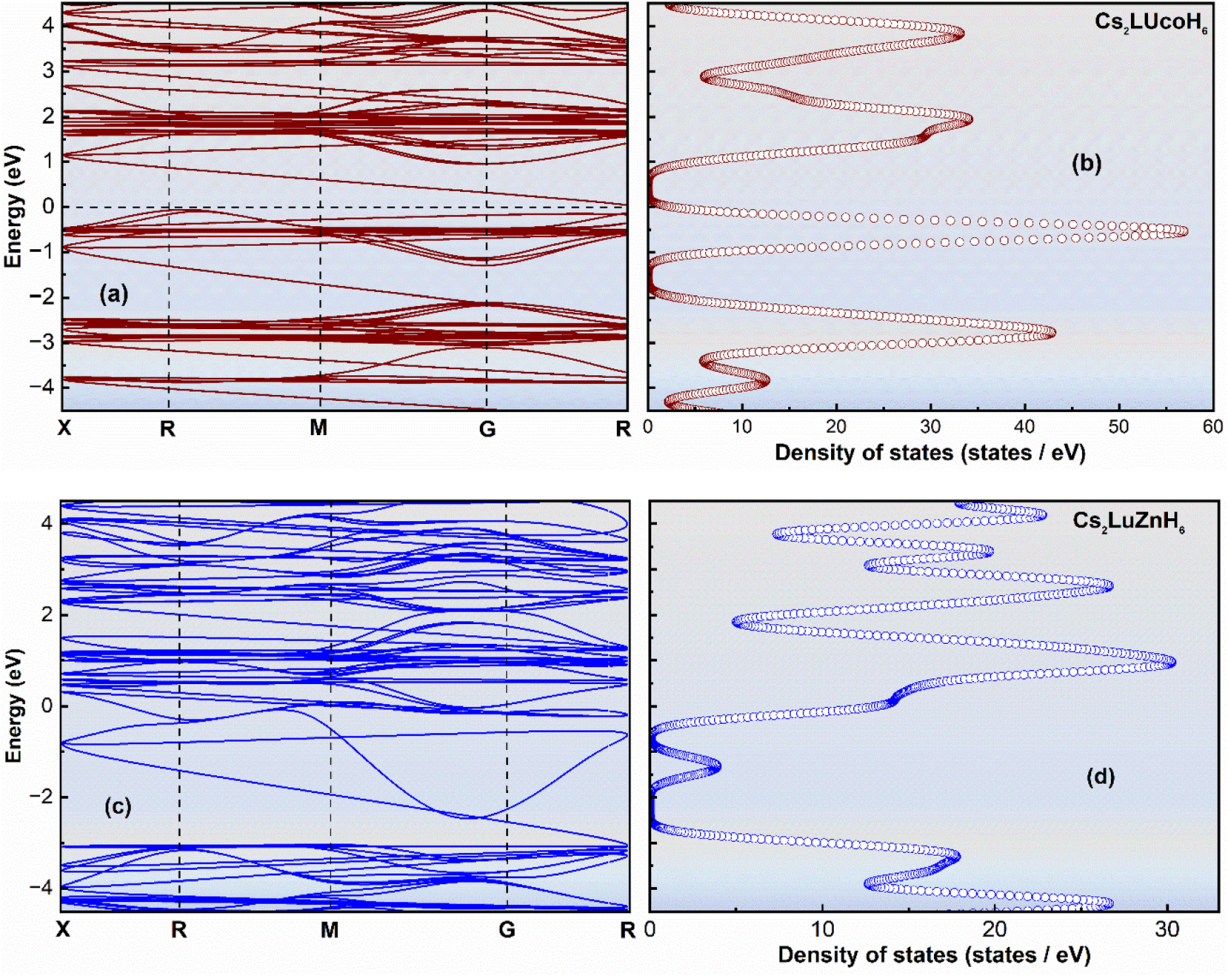
The computed plots for the energy bands (a and c) and DOS (b and d) for hydrides Cs_2_LuCoH_6_ and Cs_2_LuZnH_6_, using the hybrid HSE06 functional.

**Fig. 3:**
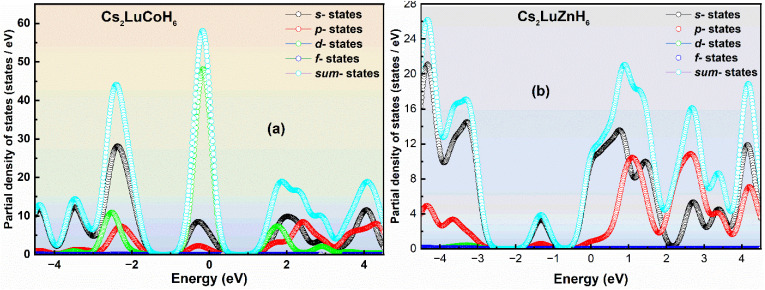
The calculated PDOS plots (a and b) for the double hydrides Cs_2_LuCoH_6_ and Cs_2_LuZnH_6_, obtained using the hybrid HSE06 functional.

The electronic properties, including the band structure, DOS, and PDOS, play a key role in determining the material behavior and its potential applications across various fields.^30^ The electronic properties actually determine the material's response to electrical influence, affecting its behavior in various ways, such as its conductivity, optical properties, device performance, energy applications and use in materials science. The band structure illustrates the range of energy levels that an electron can occupy in the solid material. By calculating the energy band gap, we can predict the type of material: conductor, semiconductor or insulator.^31,32^ The calculated band structure, DOS, and PDOS of the double hydride systems Cs_2_LuCoH_6_ and Cs_2_LuZnH_6_ are calculated by applying the complex hybrid HSE06 functional, and the results are illustrated in Fig. 2(a–d) and in Fig. 3(a and b). From Fig. 2(a) and (c) it is observed that in Cs_2_LuCoH_6_, a very narrow energy band gap of 0.917 eV exists, indicating the semiconductor behavior of the material. However, in Cs_2_LuZnH_6_, the conduction and valence energy bands in the band structure overlap with zero band gap, illustrating the metallic behavior of the material. The shape of the band structure is different in each material due to the different cations, *i.e.*, Co or Zn, in Cs_2_LuCoH_6_ and Cs_2_LuZnH_6_.

Furthermore, the DOS illustrates the number of available electronic states per unit energy interval in a given compound. The DOS plots give valuable insights into material behavior and properties.^33^Fig. 2(b) and (d) represent the DOS plots for Cs_2_LuCoH_6_ and Cs_2_LuZnH_6_ double hydride materials, predicting the behavior of these compounds. The maximum peaks of the DOS have been observed in the conduction energy bands as 34.52 states per eV at 1.97 eV and 33.52 states per eV at 3.86 eV, along with peaks in the valence energy bands of 57.58 states per eV at −0.57 eV and 43.31 states per eV at −2.76 eV in the Cs_2_LuCoH_6_ material, as shown in Fig. 2(b). Similarly, the maximum values of DOS have been noted in the valence energy bands as 30.52 states per eV at 0.98 eV and 36.97 states per eV at 2.65 eV, along with peaks in the valence energy bands of 17.98 states per eV at −3.28 eV and 26.87 states per eV at −4.35 eV in the Cs_2_LuZnH_6_ material, as shown in Fig. 2(d).

The concept of PDOS in solid-state physics explains the contribution of each specific atomic state to the overall electronic DOS in the compound under consideration. A PDOS examination highlights how individual atomic orbitals contribute to the electronic properties.^34,35^ The PDOS for the Cs_2_LuCoH_6_ and Cs_2_LuZnH_6_ double hydride perovskite materials are calculated, and the results for the PDOS are illustrated in Fig. 3(a and b). The electronic configurations of Cs, Lu, Co, Zn, and H in Cs_2_LuCoH_6_ and Cs_2_LuZnH_6_ perovskites are listed as 5s^2^ 5p^6^ 6s^1^, 5s^2^ 5p^6^ 5d^1^ 6s^2^, 3s^2^ 3p^6^ 3d^7^ 4s^2^, 3p^6^ 4s^2^ 3d^10^, and 1s^1^, respectively. This means that 6s^1^ of Cs, 5d^1^ of Lu, 3d^7^ of Co, and 1s^1^ of H make major contributions to the PDOS plots for Cs_2_LuCoH_6_. The peak values are listed for s-states as 28.68 states per eV at −2.34 eV, for p-states as 8.91 states per eV at −2.42 eV, for d-states as 48.73 states per eV at −0.19 eV, and for sum-states as 58.78 states per eV at −0.17 eV for the Cs_2_LuCoH_6_ material, as represented in Fig. 3(a). 6s^1^ of Cs, 5d^1^ of Lu, and 1s^1^ of H make major contributions to the PDOS plots for Cs_2_LuZnH_6_. The maximum values for Cs_2_LuZnH_6_ material are 13.83 states per eV at 0.74 eV for s-states,10.72 states per eV at 1.08 eV for p-states, and 21.52 states per eV at 0.88 eV for sum-states, as represented in Fig. 3(b).

**Fig. 4:**
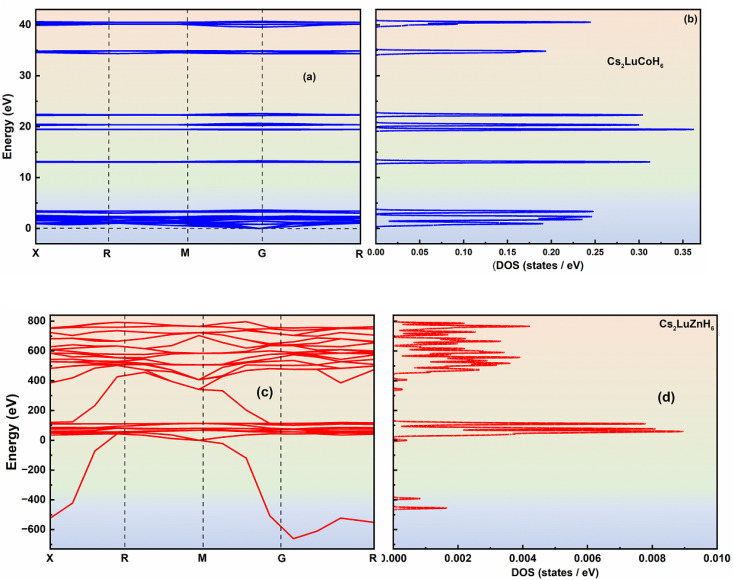
Simulated plots showing the dispersion curves (a and c) and DOS (b and d) for Cs_2_LuCoH_6_ and Cs_2_LuZnH_6_, respectively, using the HSE06 hybrid functional.

Phonons act as the primary carriers of vibrational energy in the materials under investigation. The allowed frequency modes in the primitive lattice cell of the crystal of the material for the propagation of vibrational waves can be separated into two portions. The lower and upper branches are identified as acoustic and optical sections, respectively. At low frequency, the wavelength of acoustic phonons becomes large, and they behave as a sound wave in the primitive cell of the material crystal.^36^ The longitudinal and transverse phonons in the structural arrangement of the studied materials are abbreviated as LA and TA, respectively. Few non-zero minimum frequencies for optical phonons, even at very large wavelengths, still exist. In this regard, there is an interaction between the incident radiation and optical phonons of the material, termed as the infra-active region in the phonon dispersion curves.^37^ Raman scattering between incident radiation and optical phonons is termed as the Raman active region. The longitudinal and transverse phonons here are abbreviated as LO and TO, respectively. The dynamics, vibrations, stability and compatibility of a considered material are verified based on the phonon dispersion curves. Phonon dispersion with real (positive) modes of phonons confirms the vibrational and dynamic stability of the considered material.^38^ Phonon dispersion graphs and DOS for Cs_2_LuCoH_6_ and Cs_2_LuZnH_6_ double hydride perovskites are plotted by constructing a 1 × 2 × 1 supercell. Fig. 4(a–d) predicts the results for the phonon dispersion relations and the DOS for these double hydride perovskites. Fig. 4(a) and (c) illustrate that real (positive) modes of phonons are observed for Cs_2_LuCoH_6_ with no negative (imaginary) modes of phonons. However, in the case of the Cs_2_LuZnH_6_ perovskite, as shown in Fig. 4(b), a few imaginary phonons are observed, but a majority of real (positive) phonons are observed in the phonon dispersion relationship. The results obtained from the phonon dispersion curves illustrate the dynamic and vibrational compatibility, reliability, and stability of these Cs_2_LuCoH_6_ and Cs_2_LuZnH_6_ hydride perovskites for use in hydrogen storage devices. Fig. 4(b) and (d) illustrates the DOS for the phonons of Cs_2_LuCoH_6_ and Cs_2_LuZnH_6_ hydride perovskites. It is observed that no phonon states are observed in the negative (imaginary) region around the Fermi level of the DOS for the Cs_2_LuCoH_6_ perovskite material. The highest DOS peak for Cs_2_LuCoH_6_ of 0.36 states per eV at 19.27 eV is represented in Fig. 4(b). However, there are some states per eV in the imaginary (negative) region of the DOS for Cs_2_LuZnH_6_, but the maximum peaks are observed in the positive (real) region of the DOS, with maximum peaks listed at 0.009 states per eV at 61.33 eV, 0.008 states per eV at 81.33 eV, and 0.007 states per eV at 113.33 eV, as shown in Fig. 4(d). Finally, the results of DOS for the phonons again confirmed that Cs_2_LuCoH_6_ and Cs_2_LuZnH_6_ have vibrational and dynamical stability, rendering these materials suitable for hydrogen storage technologies and associated applications.

The magnetic behavior of a material describes its response to an external magnetic field and is fundamentally governed by the spin-dependent electronic structure. In functional materials for hydrogen energy storage and related applications, magnetic properties are particularly important as they provide insight into electronic exchange interactions, spin polarization, and orbital contributions, which can significantly influence the overall electronic stability and transport behavior.^39^

**Fig. 5:**
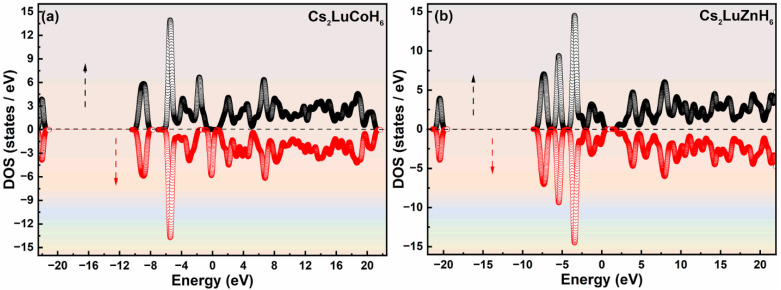
The computed spin-resolved DOS spectra for (a) Cs_2_LuCoH_6_ and (b) Cs_2_LuZnH_6_, obtained by utilizing the HSE06 functional.

**Fig. 6:**
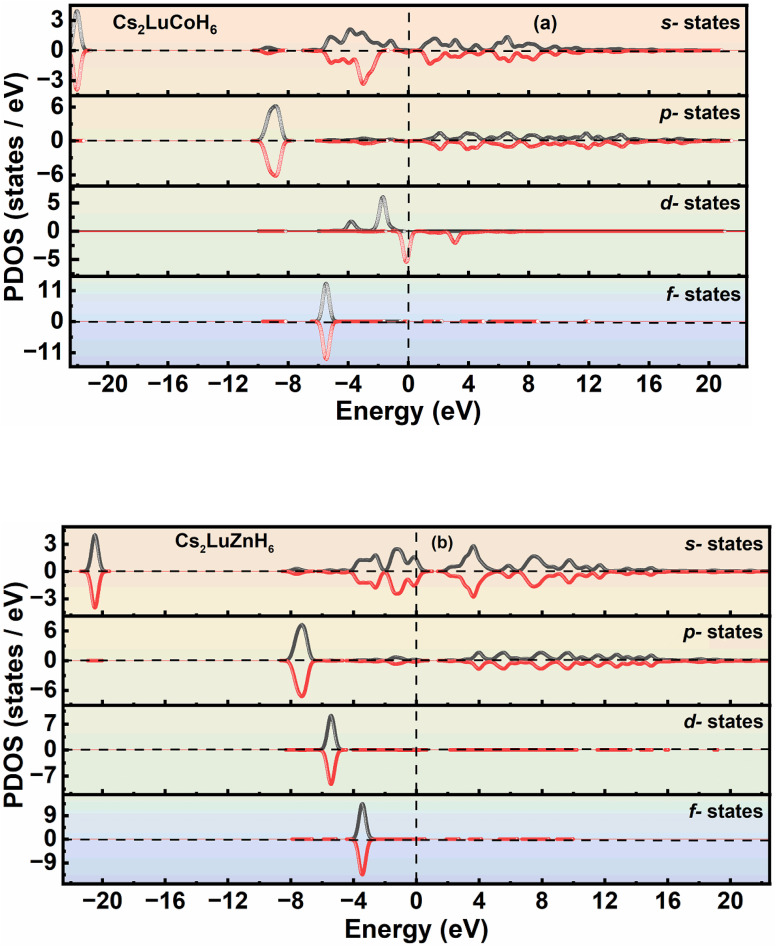
The calculated graphs for spin-polarized PDOS for (a) Cs_2_LuCoH_6_ and (b) Cs_2_LuZnH_6_, utilizing the HSE06 functional.

In the present study, the magnetic properties of Cs_2_LuCoH_6_ and Cs_2_LuZnH_6_ were investigated through DOS and PDOS calculations using the HSE06 hybrid functional, as shown in Fig. 5a, b and 6a, b, respectively.

For Cs_2_LuCoH_6_, the spin-resolved DOS exhibits clear asymmetry between spin-up (↑) and spin-down (↓) channels near the Fermi level (set at 0 eV). This spin polarization arises primarily from the partially filled Co-3d orbitals, which undergo exchange splitting and contribute unequally to the two spin channels. As a result, a net magnetic moment of 1.25 µB is obtained, confirming a ferromagnetic ground state. The PDOS further reveals strong hybridization between Co-3d and H-1s states, which contributes to magnetic ordering through indirect exchange interactions.^40^

In contrast, Cs_2_LuZnH_6_ exhibits a completely symmetric spin-up and spin-down DOS, indicating no spin polarization and a non-magnetic ground state. This behavior originates from the fully filled Zn-3d^10^ electronic configuration, where the Zn-3d states lie deep below the Fermi level and do not contribute to exchange splitting. Consequently, both spin channels are identical, leading to a zero net magnetic moment (0 µB), as confirmed by DOS and PDOS analysis.

These results determine that the magnetic behavior in Cs_2_LuCoH_6_ is driven by partially filled transition-metal Co-3d states, whereas the absence of unpaired d-electrons in Zn leads to non-magnetic behavior in Cs_2_LuZnH_6_.

In addition, the spin-polarized PDOS profiles as a function of frequency for Cs_2_LuCoH_6_ and Cs_2_LuZnH_6_ double hydride perovskites have also been explored to verify the magnetic properties as predicted by DOS for these materials. The results showing the PDOS for Cs_2_LuCoH_6_ and Cs_2_LuZnH_6_ are presented in Fig. 6(a and b). From Fig. 6(a), it is observed that the spin-down ↓ and spin-up ↑ states of s-states in the valence band and the d-state located at the Fermi energy make contributions to the magnetic behavior of the Cs_2_LuCoH_6_ material. Also, there is an exact mirror reflection of the spin-down ↓ and spin-up ↑ states of p-states and f-states in the PDOS plots for the Cs_2_LuCoH_6_ material. The PDOS plots confirmed the magnetic behavior of the Cs_2_LuCoH_6_ material with a magnetic dipole moment of 1.25 Bohr. Furthermore, the PDOS plots of the Cs_2_LuZnH_6_ material have also been calculated, and the results are presented in Fig. 6(b). It is observed that there is an exact mirror reflection of the spin-down ↓ and spin-up ↑ states for the s-states, p-states, d-states and f-states in the PDOS plots for the Cs_2_LuZnH_6_ material. From the exact replicas of the spin-down ↓ and spin-up ↑ states in Fig. 4(b), it is confirmed that the Cs_2_LuZnH_6_ material has nonmagnetic behavior with a zero magnetic dipole moment.

The mechanical parameters of a compound illustrate its ability to withstand applied mechanical forces and deformations under various loading conditions. These properties provide insight into the compound's integrity, defect behavior, microstructural integrity and internal bonding strength. Physically, these properties arise from crystal structure, microstructural, and atomic bonding features, such as dislocations, phase compositions and grain boundaries.^41^ The most commonly evaluated properties include ductility, fracture toughness, strength, hardness, and impact resistance. In this study, the mechanical properties of Cs_2_LuCoH_6_ and Cs_2_LuZnH_6_ double hydride perovskite compounds were calculated *via* the HSE06 hybrid functional. The intrinsic elastic stiffness parameters *C*_12_, *C*_11_ and *C*_44_ were thus calculated for these hydride perovskites under an applied mechanical load. Born defined the elastic stability criteria as follows:^42^

2 *C*_44_ > 0, *C*_11_ > *C*_12_ and *C*_11_ + 2*C*_12_ > 0

Stiffness mechanical parameters such as bulk modulus (*B*), Voigt shear modulus (*G*_v_), Reuss isotropic shear modulus (*G*_r_), shear modulus (*G*), and Young's modulus (*Y*) are calculated *via* Voigt–Reuss–Hill and are represented by the given relationships:^43^3
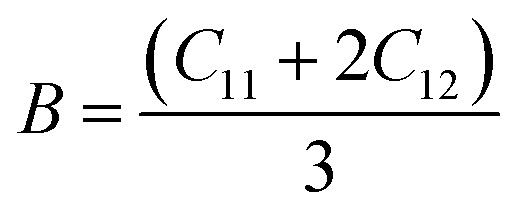
4
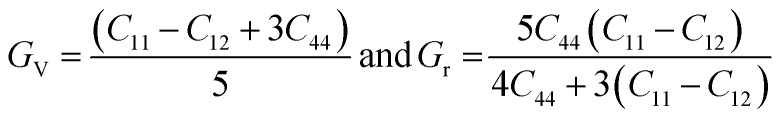
5
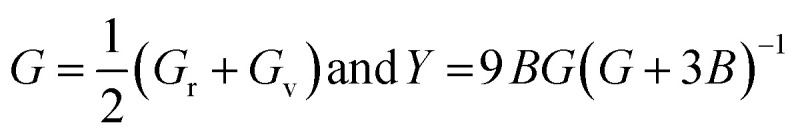


Poisson's ratio (*ν*) and the anisotropy factor (*A*) are dimensionless parameters used to describe the elastic and mechanical properties of a material, such as plasticity, anisotropy, and stiffness, and are defined by the following relationships:^44^6
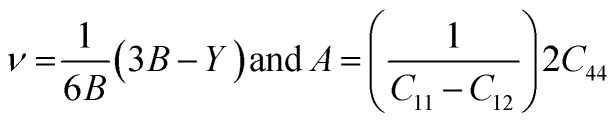


For a given material, the melting temperature in terms of elastic mechanical constants is expressed by the following relationship:

7 *T*_melt_(K) = (553 + *C*_12_ 5.922) ± 300 K

**Fig. 7:**
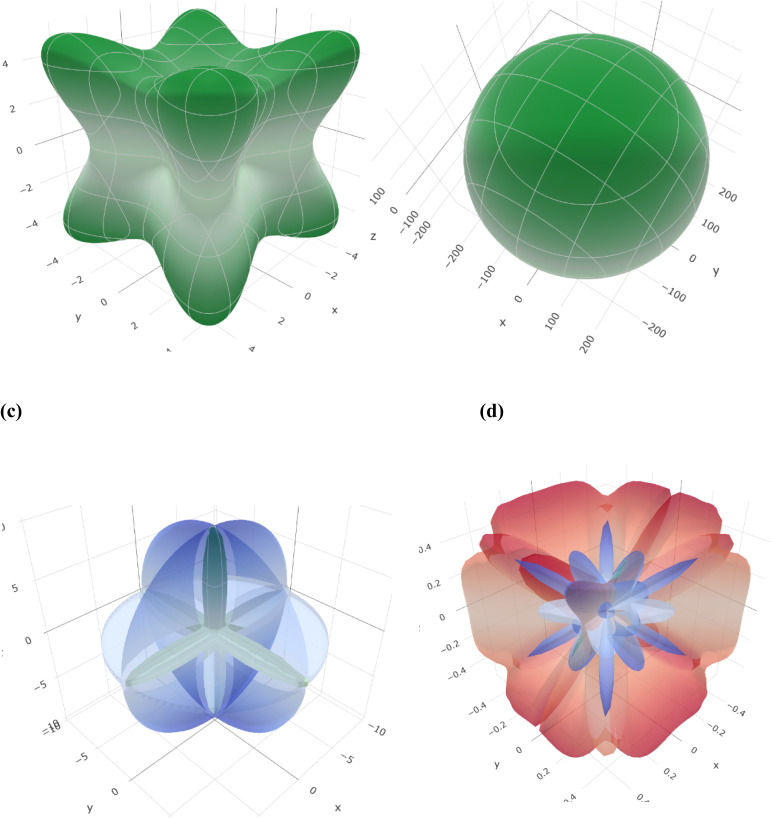
3D visualizations of mechanical properties: (a) Young's modulus, (b) linear compressibility, (c) shear modulus, and (d) Poisson's ratio.

Table 1 shows the calculated mechanical parameters for the hydride perovskites Cs_2_LuCoH_6_ and Cs_2_LuZnH_6._ The listed values include the elastic stiffness constants *C*_11_, *C*_12_, and *C*_44_ (in GPa), Poisson's ratio (*ν*), Cauchy pressure (*C*_p_) in GPa, and Pugh's ratio (*B*/*G*). Additionally, the table summarizes the bulk modulus (*B*), compressibility (*β*) in GPa, Young's modulus (*Y*), anisotropy factor (*A*), shear modulus (*G*), and the estimated melting temperature (*T*_melt_) in K, providing a comprehensive overview of the mechanical stability and elastic behavior of these hydride perovskite materials. The bulk modulus of a material quantifies its resistance to uniform compressive stress, reflecting the strength of the interatomic bonds. The trend of the calculated bulk modulus values is given as Cs_2_LuCoH_6_ (3.03 GPa) > Cs_2_LuZnH_6_ (0.52 GPa). The deviation in the calculated values of Young's modulus is listed as Cs_2_LuCoH_6_ (35.26 GPa) > Cs_2_LuZnH_6_ (6.67 GPa). The determined values of Young's modulus illustrate that Cs_2_LuCoH_6_ and Cs_2_LuZnH_6_ are softer materials, as their calculated values are less than 40 GPa, which predicts their value for use in hydrogen storage systems and associated applications. Furthermore, the Poisson's ratio (*ν*) is an important parameter in terms of mechanical properties to distinguish between ductile and brittle studied materials. A material exhibits predominantly brittle nature when *ν* is less than 0.26, whereas for other values of *ν*, ductile behavior is prominent. The calculated values for the Poisson ratio (*ν*) are listed as Cs_2_LuCoH_6_ (2.44) > Cs_2_LuZnH_6_ (1.64). The determined values for *ν* illustrate that Cs_2_LuCoH_6_ and Cs_2_LuZnH_6_ possess ductile behavior, as *ν* > 0.26.^45^ The additional mechanical property of Pugh's ratio (*B*/*G*) is also used to identify the ductile or brittle behavior of a studied material. The critical threshold for the *B*/*G* ratio is 1.75. A ductile nature is dominant for a material when *B*/*G* is greater than 1.75, whereas for other values, brittle behavior is prominent. The computed values for *B*/*G* follow the trend Cs_2_LuZnH_6_ (1.82) > Cs_2_LuCoH_6_ (4.00). These values of *B*/*G* confirm the ductile nature of the Cs_2_LuCoH_6_ and Cs_2_LuZnH_6_ materials. The inherent ductility of Cs_2_LuCoH_6_ and Cs_2_LuZnH_6_ enhances their suitability for hydrogen storage by improving resistance to cracking, accommodating defects, and enabling practical use in storage tanks and pipelines. The anisotropy factor is a crucial parameter for judging the isotropic or anisotropic behavior of a studied material. Isotropic behavior is dominant for *A* ≥ 1, whereas for other values, anisotropic behavior is prominent in a material.^46–48^ The calculated trend for the anisotropy factor is listed as Cs_2_LuCoH_6_ (−7.73) > Cs_2_LuZnH_6_ (−9.76). The determined values predict an anisotropic nature for the Cs_2_LuCoH_6_ and Cs_2_LuZnH_6_ materials. In conclusion, these mechanical properties predict that these materials are novel and transformative and should motivate experimental researchers to synthesize these compounds for hydrogen storage and related applications. Fig. 7(a–d) shows the 3D visualizations of the mechanical properties of Young's modulus, linear compressibility, shear modulus, and Poisson's ratio.

**Table 1:** The computed stiffness elastic constants *C*_44_, *C*_11_, and *C*_12_ (GPa); *ν*, the Poisson coefficient; *C*_p_, the Cauchy pressure (GPa); *B*/*G*, Pugh's ratio; *B*, bulk modulus (GPa); compressibility, *β* (G Pa)^−1^; *Y*, Young's modulus (GPa); *A*, anisotropy factor; *G*, shear modulus (GPa) and *T*_melt_, melting temperature (K) for Cs_2_LuCoH_6_ and Cs_2_LuZnH_6_ hydride perovskite materials

Parameter	Cs_2_LuCoH_6_	Cs_2_LuZnH_6_
*C*_44_	10.167	9.68
*C*_11_	−6.26	2.68
*C*_12_	−1.42	0.56
*ν*	2.43	1.64
*B*/*G*	1.82	4.00
*B*	3.04	0.52
*β*	0.0000	0.0000
*Y*	35.26	6.67
*A*	−7.73	−9.76
*G*_R_	9.14	5.42
*G*_V_	5.13	−5.16
*G*	1.67	0.13
*T*_melt_	858.92	837.75

The gravimetric H storage capacity, also known as the hydrogen weight percent or gravimetric hydrogen density, is defined as the fraction of hydrogen mass that can be released or stored by a material under consideration. The gravimetric H storage capacity represents the fraction of hydrogen mass as a percentage of the total material mass. As hydrogen is a low-cost and lightweight element, materials with large gravimetric ratios are considered the best materials, as these materials can store large amounts of hydrogen without much extra mass.^49^ Materials with a large gravimetric ratio have more energy per unit mass, making these materials crucial for many mobile applications like drones, fuel cells for vehicles, and portable devices. The gravimetric ratio for hydrogen storage materials is calculated by the given relationship:^50^8
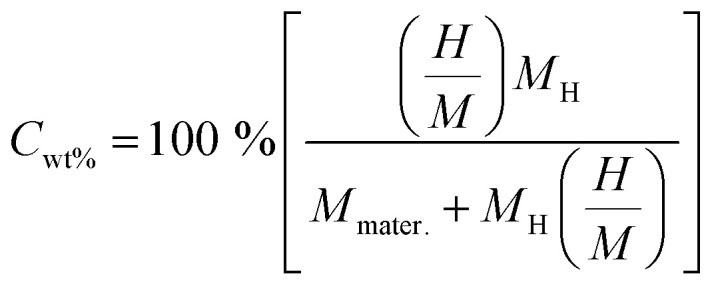
where the equation shows the ratio of H to material mass, *M*_H_ is the mass of hydrogen, and *M*_mater._ is the mass of the material under consideration. The determined trend in gravimetric ratio for the double hydride perovskites is Cs_2_LuCoH_6_ (4.87 wt%) > Cs_2_LuZnH_6_ (4.46 wt%). The simulated values of the gravimetric ratio for Cs_2_LuCoH_6_ and Cs_2_LuZnH_6_ exhibit excellent agreement with previously reported results by Hayat *et al.*^51^ for similar hydride materials.

The sufficiently large gravimetric ratios illustrate that these double hydride perovskites possess capable hydrogen storage capabilities. Therefore, Cs_2_LuCoH_6_ and Cs_2_LuZnH_6_ can be considered as potential candidates for the design and computational modeling of compounds for hydrogen-based energy storage and fuel cell applications.

### Tolerance factor

The tolerance factor (*τ*), commonly referred to as Goldschmidt's tolerance factor, is an important dimensionless parameter used to assess the structural stability and physical characteristics of perovskite materials.^52,53^ It helps predict whether compounds with compositions such as A_2_BB′X_6_ or ABX_3_ are likely to crystallize in cubic, distorted, symmetric, stable, or non-perovskite phases before performing computational optimization or experimental synthesis. The expression for *τ* is given as follows:^54,55^9
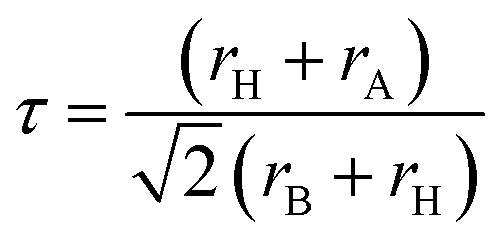
In the above expression, *r*_A_ and *r*_H_ are the radii of the A and H cations. The radius of the B cation is given as 
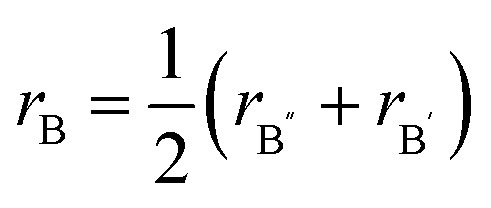
 for a double hydride A_2_B^′^B^″^H_6_ perovskite material.

For perovskite-type hydride materials, the tolerance factor (*τ*) typically exists within the range of 0.8 ≤ *τ* ≤ 1.0. Moreover, a *τ* value between 0.9 and 1.0 matches an ideal and highly symmetric cubic hydride perovskite crystal structure.^56–59^ The determined *τ* values for the considered hydride double compounds illustrate the trend Cs_2_LuCoH_6_ (0.92) > Cs_2_LuZnH_6_ (0.84). These results predict that Cs_2_LuCoH_6_ possesses nearly perfect perovskite hydride geometry, while Cs_2_LuZnH_6_ shows slight crystal structural distortion. The confirmed suitable tolerance factors verified the structural feasibility and stability of these compounds within the framework of perovskite structures. These results illustrate that Cs_2_LuCoH_6_ and Cs_2_LuZnH_6_ are promising and game-changing materials for simulations and for the synthesis of hydrogen energy fuel cells and many other clean hydrogen storage systems.

## Conclusions

Large calculated gravimetric ratios of 4.87 wt% (Cs_2_LuCoH_6_) and 4.46 wt% (Cs_2_LuCoH_6_) are observed for these materials. The calculated mechanical parameters include bulk modulus, where Cs_2_LuCoH_6_ (3.03 GPa) > Cs_2_LuZnH_6_ (0.52 GPa); Young's modulus, where Cs_2_LuCoH_6_ (35.26 GPa) > Cs_2_LuZnH_6_ (6.67 GPa); Poisson's coefficient (*ν*), where Cs_2_LuCoH_6_ (2.44) > Cs_2_LuZnH_6_ (1.64); and anisotropy factor, where Cs_2_LuCoH_6_ (−7.73) > Cs_2_LuZnH_6_ (−9.76). The metallic character of Cs_2_LuZnH_6_ and the semiconductor character of Cs_2_LuCoH_6_ are observed from the electronic properties. The non-magnetic behavior of Cs_2_LuZnH_6_ and magnetic behavior of Cs_2_LuCoH_6_ with a magnetic moment of 1.25 Bohr are predicted from the magnetic properties of these materials. The positive modes of phonons for Cs_2_LuCoH_6_, with no negative modes, but with a few negative modes for Cs_2_LuZnH_6_, are observed in the phonon dispersion graphs. Finally, the results of physical property analysis, such as electronic, vibrational, magnetic and mechanical properties, predict that Cs_2_LuCoH_6_ and Cs_2_LuZnH_6_ are potential and game-changing materials for hydrogen storage devices and related applications.

## Conflicts of interest

The authors declare that they have no known competing financial interests or personal relationships that could have appeared to influence the work reported in this paper.

## Data Availability

All data that support the findings of this study are included with the article.
